# Pleiotropic Actions of FGF23

**DOI:** 10.1177/0192623317737469

**Published:** 2017-11-02

**Authors:** Reinhold G. Erben

**Affiliations:** 1 Department of Biomedical Sciences, Institute of Physiology, Pathophysiology and Biophysics, University of Veterinary Medicine, Vienna, Austria

**Keywords:** endocrine system, renal, transgenic animals, bone, cardiovascular system, mineral metabolism, bone mineralization

## Abstract

Fibroblast growth factor-23 (FGF23) is a bone-derived hormone, mainly produced by osteoblasts and osteocytes in response to increased extracellular phosphate and circulating vitamin D hormone. Endocrine FGF23 signaling requires co-expression of the ubiquitously expressed FGF receptor 1 (FGFR1) and the co-receptor α-Klotho (Klotho). In proximal renal tubules, FGF23 suppresses the membrane expression of the sodium–phosphate cotransporters Npt2a and Npt2c which mediate urinary reabsorption of filtered phosphate. In addition, FGF23 suppresses proximal tubular expression of 1α-hydroxylase, the key enzyme responsible for vitamin D hormone production. In distal renal tubules, FGF23 signaling activates with-no-lysine kinase 4, leading to increased renal tubular reabsorption of calcium and sodium. Therefore, FGF23 is not only a phosphaturic but also a calcium- and sodium-conserving hormone, a finding that may have important implications for the pathophysiology of chronic kidney disease. Besides these endocrine, Klotho-dependent functions of FGF23, FGF23 is also an auto-/paracrine suppressor of tissue-nonspecific alkaline phosphatase transcription via Klotho-independent FGFR3 signaling, leading to local inhibition of mineralization through accumulation of pyrophosphate. In addition, FGF23 may target the heart via an FGFR4-mediated Klotho-independent signaling cascade. Taken together, there is emerging evidence that FGF23 is a pleiotropic hormone, linking bone with several other organ systems.

## Introduction

Intact fibroblast growth factor-23 (FGF23) circulating in the bloodstream is a 32-kDa glycoprotein consisting of 227 amino acids. FGF23 was discovered as a novel member of the FGF family in the year 2000, when mutations putatively interfering with cleavage of the protein were identified as the cause of autosomal dominant hypophosphatemic rickets (ADHRs), an inherited renal phosphate-wasting disease (The ADHR Consortium 2000). Although more or less at the same time FGF23 was also described in thalamic nuclei of the murine brain (Yamashita, Yoshioka, and Itoh 2000), the seminal discovery of a link between FGF23 and phosphate-wasting diseases made by the ADHR consortium opened up an exciting new field of research.

It is now firmly established that FGF23 suppresses the abundance of phosphate-transporting molecules in the apical membrane of epithelial cells in the proximal renal tubule, leading to reduced reabsorption of phosphate from the urine (Shimada, Hasegawa, et al. 2004; Shimada et al. 2005). In addition, FGF23 signaling suppresses proximal tubular expression of 1α-hydroxylase, the rate-limiting step in vitamin D hormone production, thereby reducing blood concentrations of the active vitamin D hormone, 1α,25-dihydroxyvitamin D_3_, 1,25(OH)_2_D_3_ (Shimada, Hasegawa, et al. 2004; Shimada et al. 2001; Shimada et al. 2005). Only the intact FGF23 molecule is biologically active. FGF23 is inactivated by proteolytic cleavage at a conserved furin cleavage site within the FGF23 protein, which is mutated in ADHR patients. In subjects with normal kidney function, increased blood concentrations of intact FGF23 lead to renal phosphate wasting and subsequently impaired bone mineralization. Increased serum concentrations of intact FGF23 are a hallmark of renal phosphate-wasting diseases such as ADHR, X-linked hypophosphatemia (XLH), tumor-induced osteomalacia, or autosomal recessive hypophosphatemic rickets 1 (Martin, David, and Quarles 2012). Another elimination pathway for FGF23 is ultrafiltration in the kidney. FGF23 is a small 32-kD protein and therefore filtered and degraded in the kidney. The exact extent of filtration is not known, but experimental nephrectomy leads to a rapid increase in circulating FGF23 (Mace et al. 2015). Therefore, it is likely that reductions in glomerular filtration rate per se lead to concomitant increases in circulating FGF23 concentration.

FGF23 belongs to the group of endocrine FGFs (Itoh, Ohta, and Konishi 2015). FGF23 is found in all vertebrates (Itoh, Ohta, and Konishi 2015). Most members of the gene family of FGFs are paracrine or intracellular signaling molecules. However, the 3 endocrine FGFs, FGF19, FGF21, and FGF23, reach their target tissues via the blood stream. Endocrine FGFs lack a functional heparan sulfate–binding site which is characteristic of canonical FGFs. All FGFs signal through 4 different tyrosine kinase receptors, namely, FGFR1, 2, 3, and 4. Because heparan sulfate enhances binding of paracrine FGFs to FGFRs at the cell surface, high affinity binding of endocrine FGFs to FGFRs requires the co-receptor proteins α- and β-Klotho (Urakawa et al. 2006; Goetz et al. 2007; Goetz, Ohnishi, Ding, et al. 2012; Goetz, Ohnishi, Kir, et al. 2012). α- and β-Klotho are transmembrane proteins expressed in target cells of endocrine FGFs. α-Klotho increases the binding affinity of FGF23 to FGFR1 by a factor of about 20, whereas binding of FGF19 and 21 to FGFRs is enhanced by β-Klotho (Goetz, Ohnishi, Ding, et al. et al. 2012; Goetz, Ohnishi, Kir, et al. 2012).

During recent years, it has been discovered that FGF23 is not only a phosphaturic hormone but also regulates calcium and sodium uptake in the kidney (Andrukhova, Slavic, et al. 2014; Andrukhova, Smorodchenko, et al. 2014; Han et al. 2016). Moreover, novel Klotho-independent actions of FGF23 were described in the heart (Faul et al. 2011; Grabner et al. 2015), bone (Murali, Roschger, et al. 2016), and immune cells (Rossaint et al. 2016). Thus, FGF23 has recently emerged as a pleiotropic hormone, linking bone with various other organ systems. The purpose of this review is to provide a concise overview over the recent advances in this area.

## Regulation of FGF23 Secretion

Under physiological circumstances, the major cellular sources of FGF23 circulating in the bloodstream are osteoblasts and osteocytes in bone (Yoshiko et al. 2007; Martin, David, and Quarles 2012). However, under pathological conditions, other cell types such as immune cells in systemic inflammatory processes (Masuda et al. 2015) or cardiomyocytes after experimental myocardial infarction or in patients with left ventricular hypertrophy (Andrukhova et al. 2015; Leifheit-Nestler et al. 2016) may become relevant sources of circulating FGF23.

The mechanisms that regulate FGF23 secretion in bone cells are only partially known. Lessons from genetic diseases such as XLH and AHRH1 have taught that disturbances in the mineralization of the organic matrix surrounding FGF23-producing cells can be a strong stimulus for their FGF23 secretion (Feng et al. 2006). Therefore, there must be some sensing mechanism for proper mineralization in bone cells. However, the nature of this sensing mechanism and the biological logic behind augmented FGF23 secretion in response to impaired mineralization have remained elusive thus far. FGFR1 signaling may be involved in the regulation of FGF23 secretion and/or the mineralization sensing mechanism because bone-specific ablation of *Fgfr1* partially rescues the excessive FGF23 secretion in *Hyp* mice, the murine homolog of XLH (Xiao et al. 2014). Conversely, gain-of-function mutations in *FGFR1* in patients with osteoglophonic dysplasia can cause increased bony FGF23 secretion (White et al. 2005).

**Figure 1.**
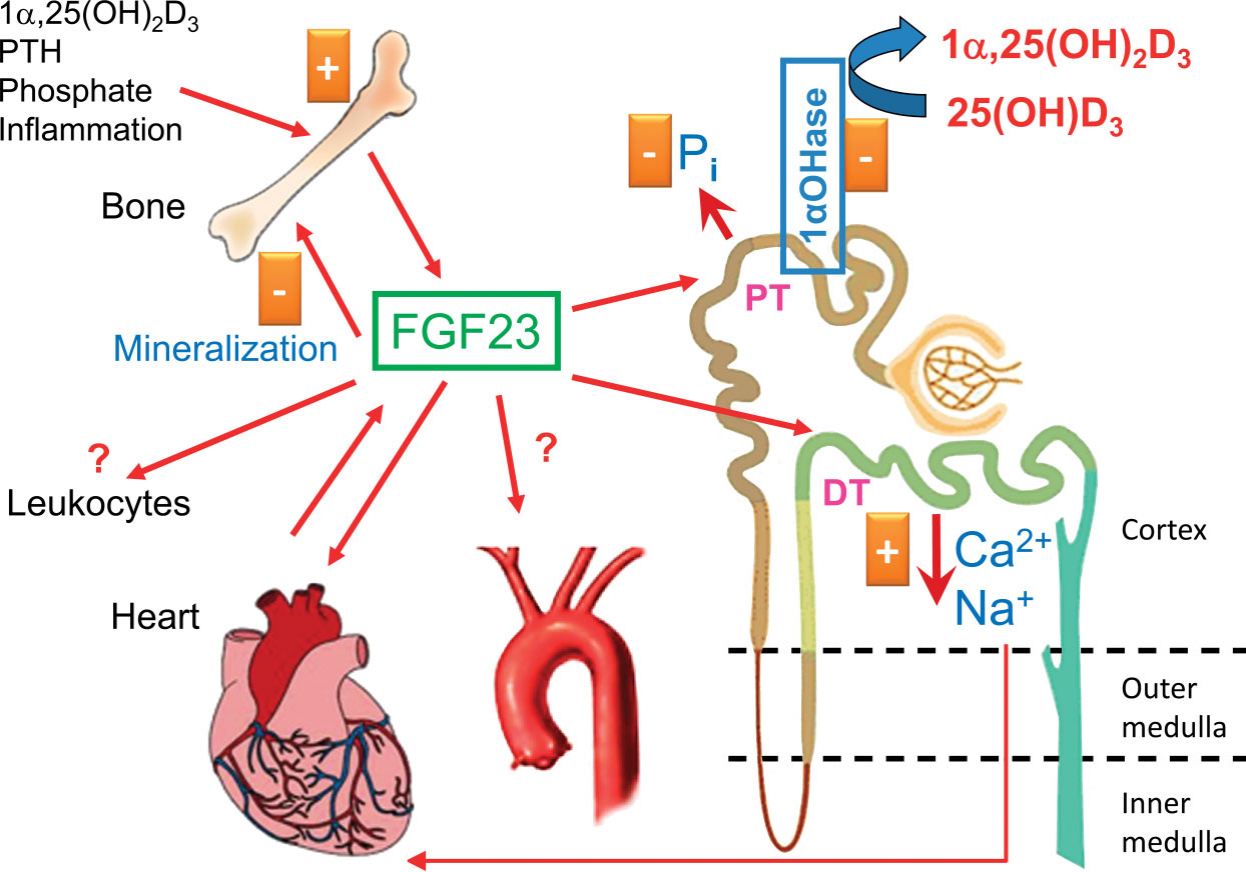
Pleiotropic actions of fibroblast growth factor-23 (FGF23). FGF23 is mainly produced in bone by osteoblasts and osteocytes under physiological conditions. Secretion of FGF23 is stimulated by phosphate, parathyroid hormone (PTH), inflammatory cytokines, and by the vitamin D hormone, 1α,25-dihydroxyvitamin D_3_, 1α,25(OH)_2_D_3_. FGF23 acts independently on proximal and distal renal tubules. In proximal renal tubules (PT), FGF23 inhibits phosphate reuptake and expression of 1α-hydroxylase, the rate-limiting enzyme for vitamin D hormone, 1α,25(OH)_2_D_3_, synthesis. In distal tubules (DT), FGF23 increases reabsorption of calcium and sodium. Increased renal sodium reabsorption may put additional strain on the heart by volume retention and subsequent hypertension. FGF23 has been shown to act as a direct pro-hypertrophic factor in the heart. In addition, the heart may become a source of circulating FGF23 under pathological conditions such as myocardial infarction or left ventricular hypertrophy. Recent evidence suggests that FGF23 acts as an auto-/paracrine regulator of bone mineralization by suppressing tissue nonspecific alkaline phosphatase in osteocytes. It is still unclear whether circulating FGF23 has direct effects on blood vessels. Moreover, emerging evidence suggests that FGF23 may modulate the function of cells of the innate immune system. The renal actions of FGF23 require the presence of the co-receptor α-Klotho in the target cell membrane, whereas the actions of FGF23 on cardiomyocytes, bone, blood vessels, and immune cells are Klotho-independent and hence may become operative only at high local concentrations.

A plethora of humoral factors such as the vitamin D hormone (Fig. 1), phosphate, calcium, parathyroid hormone (PTH), aldosterone, iron deficiency, and pro-inflammatory cytokines has been shown to directly or indirectly stimulate osteoblastic/osteocytic FGF23 secretion (Martin, David, and Quarles 2012; Quinn et al. 2013; Wolf and White 2014; Ito et al. 2015; David et al. 2016; Pathak et al. 2016; B. Zhang et al. 2016). However, with the exception of the vitamin D hormone (Yoshiko et al. 2007; Kaneko et al. 2015) and of PTH (Meir et al. 2014), the molecular mechanisms underlying the regulatory effects of the abovementioned factors on FGF23 transcription are only incompletely understood.

A novel level of complexity in the regulation of FGF23 secretion has been introduced by the finding that an adequate equilibrium between posttranslational phosphorylation and glycosylation of FGF23 is of physiological importance (Tagliabracci et al. 2014). FGF23 is O-glycosylated within the furin cleavage site by polypeptide *N*-acetylgalactosaminyltransferase 3 (GalNT3), a process that protects FGF23 from furin-mediated cleavage during the secretory process. Conversely, phosphorylation of a serine residue near the glycosylation site by family with sequence similarity 20, member C (FAM20C), hinders O-glycosylation and, thus, favors cleavage of FGF23. Both processes are physiologically essential: loss-of-function mutations in FAM20C cause increased blood concentrations of intact FGF23 and hypophosphatemic rickets (Wang et al. 2012), whereas loss-of-function mutations in *GalNT3* result in an FGF23 deficiency-like phenotype in mice and men (Topaz et al. 2004; Kato et al. 2006; Ichikawa et al. 2009). It is conceivable that the balance between posttranslational phosphorylation and glycosylation of FGF23 may be regulated by local or humoral factors. However, currently very little is known about the validity of this hypothetical concept.

## Renal Actions of FGF23

Human genetic diseases and mouse experiments have conclusively shown that both gain and loss of function of FGF23 primarily targets the kidney. As mentioned above, FGF23 was discovered as a phosphaturic hormone. However, it took over a decade to unveil the molecular mechanism underlying the phosphaturic action of FGF23. The regulation of phosphate reabsorption in the kidney takes place in proximal tubules, which express FGFR1, 3, and 4, but only little FGFR2 (Gattineni et al. 2009; Andrukhova et al. 2012). Mouse experiments with conditional deletion of *Fgfr1* have shown that FGFR1 is probably the most important FGFR mediating the actions of FGF23 in the kidney (Han et al. 2016).

Although previously controversial, it is now well established that protein expression of full-length α-Klotho is similar in proximal and distal tubular epithelial cells (Andrukhova et al. 2012). In the current model of the phosphaturic action of FGF23 (Figure 1), blood-borne FGF23 directly targets proximal tubules by binding to the α-Klotho/FGFR1 complex (Andrukhova et al. 2012). Binding of the ligand FGF23 leads to activation of an intracellular signaling cascade involving extracellular signal-regulated kinase 1 and 2 (ERK1/2) and serum/glucocorticoid-regulated kinase-1 (SGK1). Activation of SGK1 leads to phosphorylation of the scaffolding protein Na^+^/H^+^ exchange regulatory cofactor (NHERF)-1, which then results in internalization and degradation of the sodium–phosphate cotransporters NaPi-2a and NaPi-2c (Andrukhova et al. 2012). It is interesting to note in this context that the other major phosphaturic hormone, PTH, also induces NHERF-1 phosphorylation to reduce apical membrane abundance of phosphate cotransporters, albeit through a different signaling mechanism involving protein kinase A and C (Deliot et al. 2005; Weinman et al. 2007).

Much less is known about the second action of FGF23 on proximal renal tubules, namely, the downregulation of the expression of 1α-hydroxylase, the rate-limiting enzyme in 1,25(OH)_2_D_3_ synthesis (Figure 1). Although it is clear that ERK1/2 signaling is involved in the FGF23-mediated suppression of 1α-hydroxylase (M. Y. Zhang et al. 2012; Ranch et al. 2011), the molecules downstream of ERK1/2 leading to suppression of 1α-hydroxylase transcription are currently not known. Based on the phenotype of *Fgf23-*deficient mice (Shimada, Kakitani, et al. 2004; Sitara et al. 2004) and of men with loss-of-function mutations in *FGF23* (Topaz et al. 2004; Araya et al. 2005), the suppression of renal 1α-hydroxylase activity may actually be the most important physiological function of circulating intact FGF23. In mice and men, loss of FGF23 function leads to a severe phenotype characterized by elevated circulating vitamin D hormone levels, early lethality, hypercalcemia, hyperphosphatemia, and soft tissue calcifications. Lack of the co-receptor α-Klotho causes a similar phenotype in humans and mice (Kuro-o et al. 1997; Ichikawa et al. 2007). There is very good evidence that the severe phenotype of α-*Klotho-* and *Fgf23-*deficient mice is due to uncontrolled production of 1,25(OH)_2_D_3_ and subsequent chronic hypercalcemia and hyperphosphatemia because concomitant ablation of vitamin D signaling completely rescues α-*Klotho* ^−/−^ and *Fgf23* ^−*/*−^ mice (Hesse et al. 2007; Anour et al. 2012; Streicher et al. 2012). Hence, the regulation of renal 1α-hydroxylase obviously fails in the absence of FGF23 signaling, leading to unleashed production of the active vitamin D hormone with all its untoward sequelae.

During recent years, it was uncovered that FGF23 not only targets proximal renal tubules but has additional and independent functions in distal tubular epithelium as a calcium- and sodium-conserving hormone (Figure 1). In distal renal tubules, binding of FGF23 to the α-Klotho/FGFR1 complex leads to activation of with-no-lysine kinase-4 (WNK4) through an ERK1/2-SGK1 signaling cascade (Andrukhova, Slavic, et al. 2014; Andrukhova, Smorodchenko, et al. 2014). In line with the known function of WNKs as important regulators of intracellular trafficking and activation of ion channels in distal tubular epithelium (Bazua-Valenti and Gamba 2015), we found that FGF23 signaling and subsequent WNK activation increased cellular uptake of calcium and sodium by upregulating apical membrane abundance of the epithelial calcium channel transient receptor potential vanilloid-5 and of the sodium (Na) chloride channel NCC (Andrukhova, Slavic, et al. 2014; Andrukhova, Smorodchenko, et al. 2014). In agreement with these findings, mice with a specific deletion of *Fgfr1* in distal renal tubules also showed renal calcium wasting (Han et al. 2016). It is well known that sodium reabsorption in the kidney has a major role in volume and blood pressure regulation. Therefore, these findings may have important pathophysiological implications because they may provide an explanation why circulating intact FGF23 concentrations are a strong and independent predictor of adverse outcomes in patients with chronic kidney disease (CKD; Isakova et al. 2011; Faul et al. 2011; Scialla et al. 2014).

## FGF23 as a Paracrine/Autocrine Regulator of Bone Mineralization

Recent evidence from our laboratory showed that locally secreted FGF23 may act as a regulator of bone mineralization in osteocytes in an autocrine/paracrine manner (Figure 1). We found that FGF23 is a potent suppressor of tissue-nonspecific alkaline phosphatase (*Tnap*) in osteoblasts at the transcriptional level (Murali, Roschger, et al. 2016). The signaling pathway proved to be a Klotho-independent, mainly FGFR3-mediated mechanism. Although direct experimental evidence is lacking, it is likely that the concentrations of FGF23 within the osteocyte canalicular system are much higher than in the systemic circulation. Therefore, low affinity binding of FGF23 to FGFR3 in the absence of the co-receptor α-*Klotho*, whose expression is negligible in bone (Kuro-o et al. 1997; Miyagawa et al. 2014), may result in efficient signal transduction, even under physiological circumstances. Tnap is an important regulator of bone mineralization because it cleaves pyrophosphate, a key mineralization-inhibiting molecule. Therefore, FGF23-mediated autocrine/paracrine suppression of Tnap may result in increased pyrophosphate concentrations in bone. We recently showed that this mechanism contributes to impaired mineralization in diseases characterized by increased osteocytic production of FGF23: increased osteocytic FGF23 secretion in *Hyp* mice suppressed Tnap activity in osteocytes and increased pyrophosphate concentrations in bone, which could be rescued by bone-specific deletion of *Fgf23* (Murali, Andrukhova, et al. 2016).

## Cardiovascular Actions of FGF23

Similar to bone, α-Klotho is not expressed in the myocardium at physiologically significant levels (Faul et al. 2011; Grabner et al. 2015). Together with the fact that FGF23 expression is very low in the healthy heart, these findings suggest that physiological levels of circulating FGF23 do not affect the heart. However, this situation may change in situations where FGF23 is locally produced in the heart or when circulating FGF23 levels rise significantly. In patients with CKD, serum levels of intact FGF23 can reach levels 1,000-fold above normal in advanced stages of the disease (Gutierrez 2010; Weber et al. 2003). There is good evidence from rodent models, but also from humans, that cardiomyocytes are able to produce FGF23 in the strained heart. Leifheit-Nestler and coworkers (2016) demonstrated that FGF23 expression is increased in cardiomyocytes of CKD patients with left ventricular hypertrophy (Figure 1). Similarly, FGF23 expression was shown to be increased in peri-infarct cardiomyocytes in rodent models of acute myocardial infarction (Andrukhova et al. 2015). In addition, there is evidence that at high circulating concentrations, FGF23 is a pro-hypertrophic molecule, acting directly on cardiomyocytes via a Klotho-independent, FGFR4-mediated signaling pathway (Faul et al. 2011; Grabner et al. 2015). Since increased local production of FGF23 may also be able to stimulate FGFR4-mediated signaling, FGF23 may contribute to the pathogenesis of left ventricular hypertrophy in a paracrine manner.

The question whether FGF23 has direct effects on blood vessels is a controversial issue. Some studies reported a positive association between circulating FGF23 and arterial stiffness (Mirza, Larsson, et al. 2009), total body atherosclerosis (Mirza, Hansen, et al. 2009), coronary artery calcification (Morita et al. 2015; Hu et al. 2015), and carotid atherosclerosis (Shah et al. 2015) in normal subjects, whereas other studies failed to provide evidence for a link between atherosclerosis or atherosclerotic events and serum FGF23 in CKD patients (Seiler et al. 2014; Sarmento-Dias et al. 2016). There is general agreement that both human and murine arteries lack α-Klotho expression in endothelium and vascular smooth muscle cells (Scialla et al. 2013; Lindberg et al. 2013; Mencke et al. 2015). Therefore, it is unlikely that physiological levels of circulating FGF23 are directly able to influence arterial function and vascular calcification. On the other hand, vascular calcification is a frequent finding in patients with CKD, and it is conceivable that high circulating levels of intact FGF23 such as those found in CKD patients might exert Klotho-independent effects on blood vessels. However, the majority of studies in vascular smooth muscle cells or organ cultures of arteries did not suggest a direct calcification-promoting effect of FGF23 or a direct modulating effect of FGF23 on arterial dilatory or contractile functions (Lindberg et al. 2013; Scialla et al. 2013). In contrast, other studies reported a direct inhibitory effect of FGF23 on nitric oxide–mediated vasodilation in mouse aortic rings (Silswal et al. 2014). Collectively, the evidence for a causal direct link between FGF23 and vascular calcification, atherosclerosis, or endothelial dysfunction is inconclusive and needs further investigation. In addition, it is unclear whether FGF23 may have differential actions in different vascular beds.

## Immunomodulating Effects of FGF23

Although confirmation in additional models is needed, recent evidence suggested that FGF23 may have a role in host defense and leukocyte recruitment to inflamed tissues (Rossaint et al. 2016). Acting through a Klotho-independent signaling mechanism, FGF23 was shown to inhibit activation of integrins and chemokine-activated leukocyte arrest on the endothelium by FGFR2-mediated PKA activation (Rossaint et al. 2016). In analogy to the heart and blood vessels, the Klotho-independent signaling mechanism suggests that these effects may come into play only at high circulating concentrations of intact FGF23 such as those seen in CKD patients. In addition, FGF23 was shown to enhance secretion of tumor necrosis factor-α in primary cultures of murine peritoneal macrophages (Masuda et al. 2015). Experimental studies in mice have demonstrated that strong systemic inflammatory stimuli can result in profound increases in FGF23 secretion from macrophages and dendritic cells (Masuda et al. 2015). Therefore, FGF23 does not only target but is also produced by cells of the innate immune system. It is currently unclear whether FGF23 secreted from macrophages or dendritic cells may be part of the cross talk between different cell types in the innate immune system.

## Summary

The purpose of this review is to provide a concise overview of the role of the bone-derived hormone FGF23 in various organ systems. Together with FGF19 and 21, FGF23 is a member of the group of endocrine FGFs. Endocrine FGFs signal through receptor complexes of FGF receptors with the co-receptors α- and β-Klotho. The main physiological functions of FGF23 are the suppression of phosphate reabsorption and of vitamin D hormone synthesis in the kidney. Recently, several new Klotho-dependent and independent functions of FGF23 have been uncovered. These recent advances have shown that FGF23 is not only a phosphaturic and vitamin D-regulating hormone, but rather a pleiotropic factor involved in calcium and sodium homeostasis, blood pressure regulation, development of cardiac hypertrophy, bone mineralization, and functioning of the innate immune system. Hence, FGF23 links bone with several other organ systems.
